# ICP-MS for Multiplexed Protein Determination in Extracellular Vesicles from APP/PS1 Mice Blood Serum–Application to a Zn Supplementation Pilot Study

**DOI:** 10.1021/acs.analchem.5c03630

**Published:** 2025-09-15

**Authors:** Jaime Martínez-García, Beatriz Fernández, Enol Artime, Lidia Álvarez, Héctor González-Iglesias, David Clases, Rosario Pereiro

**Affiliations:** † Department of Physical and Analytical Chemistry, 16763 University of Oviedo, Julian Clavería 8, 33006 Oviedo, Spain; ‡ 585603 Fundación de Investigación Oftalmológica (FIO), Avda. Dres. Fernández-Vega, 34, 33012 Oviedo, Spain; § Dairy Research Institute of Asturias, Spanish National Research Council (IPLA-CSIC), C. Francisco Pintado Fe, 26, 33011 Oviedo, Spain; ∥ NanoMicroLab, Analytical Chemistry, Institute of Chemistry, 27267 University of Graz, Universitätsplatz 1, 8010 Graz, Austria

## Abstract

Neurodegenerative diseases represent a significant challenge due to their complex etiology, late diagnosis, and lack of effective treatments. Extracellular vesicles (EVs) have emerged as promising carriers of disease biomarkers, especially proteins, but their low abundance in biological fluids complicates their detection. Here, we present a novel strategy for the multiplexed quantitative determination of EV-associated proteins in blood serum from APP/PS1 transgenic mice, a model of Alzheimer’s disease. The method combines inductively coupled plasma–time-of-flight mass spectrometry (ICP-ToFMS) with competitive immunoassays using metal nanocluster-labeled (AuNCs, PtNCs, IrNCs) antibodies targeting Alpha-Actinin 1 (ACTN), Galectin-3-binding protein (LG3BP), and Moesin (MSN). EVs were isolated using an optimized ultracentrifugation protocol to reduce the level of serum protein contamination. Proteomic screening identified target proteins with a known relevance to neurodegeneration, and the developed assay achieved detection limits in the low femtomolar range. The approach was applied to a pilot study on Zn supplementation in 16-month-old APP/PS1 mice, revealing sex-dependent and genotype-specific differences in protein expression but sex-independent patterns in regulatory mechanisms (especially for MSN and LG3BP). Among the studied markers, MSN levels showed statistically significant differences with Zn treatment in male homozygous mice. This work demonstrates the potential of ICP-MS for sensitive and multiplexed biomarker quantification in EVs, supporting its use in neurodegenerative research and supplementation studies.

## Graphical abstract

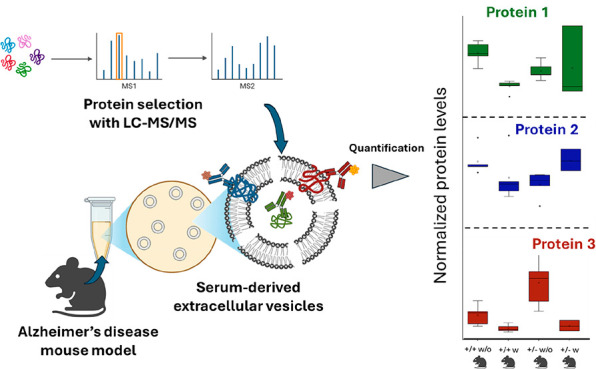

Neurodegeneration involves physiological processes in the central nervous system (CNS) that lead to diseases affecting a large portion of the aging population.^1^ Degeneration is primarily caused by the accumulation of extracellular deposits that damage neurons and glial cells, disrupt communication with the bloodstream, and ultimately lead to cell death.^2^ Current therapies for neurodegenerative diseases are not fully effective, and early diagnostic methods remain inefficient.^3,4^ Moreover, while Alzheimer’s and Parkinson’s diseases are the most extensively studied, less common disorders like Huntington’s disease and amyotrophic lateral sclerosis also contribute to diagnostic challenges due to overlapping symptoms.^5,6^ These aspects highlight the urgent need to identify disease pathways and reliable biomarkers. However, given the extremely limited access to human CNS samples, dedicated studies to study cause-effect are complicated and limit targeted development of intervention studies.^7^ Consequently, systemic body fluids like blood serum, as well as specific animal models (such as the widely used APP/PS1 mouse model for Alzheimer’s disease), are often employed in the early stages of biomarker research.^8,9^

In recent years, extracellular vesicles (EVs) have gained considerable attention regarding their potential role as biomarkers for a range of pathological conditions and for treatment options.^10^ EVs are nanometer-sized particles that carry proteins, nucleic acids, and metabolites, which can be found in any biological liquid. Proteins are relevant targets due to their role in metabolic processes and can be targeted in immunochemical approaches. However, protein levels within EVs are low and, in addition, isolating EVs from blood-derived samples is usually subjected to contamination by abundant serum proteins, leading to samples that contain unwanted proteins that may introduce artifacts.^11^ Given the limited blood volume in most common models and the low protein concentration in EVs, highly sensitive analytical methods are required to identify possible target biomarkers. In this context, inductively coupled plasma-mass spectrometry (ICP-MS) has emerged as a selective and sensitive technique for the quantitative analysis of proteins using metal-labeled immunoprobes in biological samples.^12^ Moreover, time-of-flight (ToF) MS enables the simultaneous detection of multiple analytes within a sample.^13^ Nevertheless, reports on the use of ICP-MS for EV analysis remain scarce to date. The literature describes the use of DNA sequences and antibodies labeled with lanthanides or with gold nanoparticles as recognition probes.^14–17^ Recently, Zhang et al.^18^ reported an aptamer-proximity-ligation activated rolling circle amplification method for the sensitive detection of EVs by single particle ICP-MS. This approach allowed the identification of external protein biomarkers expressed on individual EVs and the differentiation of EVs derived from tumor and normal cells.

Metal nanoclusters (MNCs), constituted of a few hundred atoms, have demonstrated a strong amplification effect enabling the detection of biomolecules at extremely low concentrations in individual cells.^19^ However, so far, their potential has not yet been explored for more challenging analysis of EVs. In this work, we present a straightforward strategy for the simultaneous determination of three proteins within EVs previously purified from serum samples of the neurodegeneration mouse model APP/PS1 using inductively coupled plasma–time-of-flight mass spectrometry (ICP-ToFMS) in combination with multiplexed competitive immunoassays based on MNC-labeled immunoprobes. The target proteins were selected through proteomic studies using liquid chromatography (LC)-MS/MS. In particular, AuNCs, PtNCs, and IrNCs were employed to label specific antibodies to Alpha-Actinin 1 (ACTN), Galectin-3-binding protein (LG3BP), and Moesin (MSN), respectively. These three proteins are closely associated with cytoskeletal dynamics and the early stages of protein aggregation, making them potentially relevant biomarkers in the context of Alzheimer’s disease. The selected proteins are located either partially (MSN) or entirely (LG3BP, ACTN) within the lumen of EVs and, therefore, a lysis protocol was implemented prior to the immunoassay to expose the EV content to MNC-labeled antibodies. The developed method was applied to the quantitative determination of targeted proteins in a pilot study of 16 month old APP/PS1 mice supplemented with zinc.

## Experimental Section

### APP/PS1 Mouse Model: Mouse Strains, Breeding, Husbandry, and Zn Supplementation

APP/PS1 double transgenic mice B6.Cg-Tg­(APPswe,PSEN1dE9)­85Dbo/­Mmjax (initially obtained from The Jackson Laboratory, JAX stock #34832) were used in the present study.^20^ APP/PS1 mice express a chimeric mouse/human amyloid precursor protein (Mo/HuAPP695swe) and a mutant human presenilin 1 (PS1-dE9), both mutations associated with early onset Alzheimer’s disease. As a result of this mutation, hemizygous transgenic mice (+/–, +/−) begin to develop beta-amyloid deposits in the brain by 6 to 7 months of age. This makes the APP/PS1 mouse a well-established and suitable *in vivo* model for Alzheimer’s disease research.

Mice were maintained on a 12 h light–dark cycle, where humidity and temperature were controlled, with food and water available *ad libitum*. All experimental procedures followed ARRIVE (Animal Research: Reporting of In Vivo Experiments) guidelines^21^ and were carried out in accordance with the European Communities Council Directive (2010/63/UE) on the protection of animals used for scientific purposes and the Spanish regulations on the protection of animals used for research. The study was approved by the Animal Research Ethics Committee of the University of Oviedo in accordance with Royal Decree 53/2013 and authorized by the Ministry of Rural Environment and Territorial Cohesion of the Government of the Principality of Asturias (PROAE 25/2021).

To investigate the potential neuroprotective effects of Zn, supplementation was carried out in the animal model by adding ZnSO_4_ monohydrate, 99% purity (ref 389802500, Thermo Scientific Chemicals), to drinking tap water to a final concentration of 30 mg L^–1^ Zn. This supplementation protocol was designed based on previous works demonstrating the beneficial role of Zn in neurodegenerative processes.^22–24^ Supplemented water was fed to APP/PS1 mice from 1-month-old until their sacrifice at 16 months. Age- and sex-matched untreated APP/PS1 mice were fed tap water. In this work, the following simplified nomenclature was adopted: +/+ for homozygous (+/+,+/+) individuals and +/– for heterozygous (+/–,+/−) individuals. Zn supplementation was indicated as w/o (without Zn) or w/w (with Zn). Each study group consisted of five animals, except for the +/– w/o Zn group, with only three mice due to limited availability.

### Instrumentation

Ultracentrifugation steps for EV isolation were performed using an Optima L-90K ultracentrifuge (Beckman Coulter, Inc.). Total protein determination was conducted via spectrophotometric measurements by using a Multiskan SkyHigh spectrophotometer (Thermo Fisher Scientific, Inc.). For the characterization of MNCs, fluorometric measurements were conducted with a PerkinElmer LS-50B luminescence spectrophotometer (PerkinElmer, Inc.). Proteomic analysis was performed using a Evosep One liquid chromatography (LC) system (Evosep) coupled to a ZenoTOF 7600 series quadrupole (Q)-ToF spectrometer (Sciex). ICP-MS measurements were carried out using two different instruments: (1) A 7900 series ICP-QMS (Agilent Technologies) equipped with a conventional Meinhard nebulizer (300 μL min^–1^) and a double pass spray chamber for MNC characterization and (2) A Vitesse ICP-ToFMS (Nu Instruments) for the multiplexed detection of target proteins in EVs purified from serum samples of the animal model. Table 1 depicts the ICP-MS operating conditions for both ICP-QMS and ICP-ToFMS instruments.

**Table 1:** ICP-QMS Operating Conditions for the Characterization of MNC Synthesis Used as Labels for the Immunoprobes and ICP-ToFMS Operating Conditions for the Analysis of Ir, Pt, and Au as Detection Labels in the Multiplexed Immunoassay Designed in This Work

	ICP-QMS	ICP-ToFMS
Acquisition mode	Spectrum	Spectrum
RF power	1550 W	1350 W
Auxiliary gas	0.9 L min^–1^	2 L min^–1^
Plasma gas	15 L min^–1^	13 L min^–1^
Nebulizer gas flow	1.07 L min^–1^	1.18 L min^–1^
Make up gas flow	0.10 L min^1^	
Collision gas (He) flow		12 mL min^–1^
Reaction gas (H_2_) flow		10 mL min^–1^
Integration time	100 ms	100 ms
Isotopes	^105^Pd (IS), ^193^Ir, ^195^Pt, ^197^Au	^185^Re (IS), ^193^Ir, ^195^Pt, ^197^Au

### Experimental Methods

#### Serum Sample Extraction

Blood was collected from anesthetized APP/PS1 mice by exsanguination through cardiac puncture at 16 months of age. After the blood sample coagulated (1 h at 37 °C followed by 6 h at 4 °C), the serum was separated by centrifugation at 2000 rpm for 5 min at 4 °C and then stored at −80 °C until further use.

#### Synthesis and Characterization of MNCs and MNC-Labeled Immunoprobes

Three different MNCs stabilized with lipoate ligands were synthesized following protocols previously reported for IrNCs, PtNCs, and AuNCs.^25–27^ Details related to the experimental procedures for the synthesis and characterization of MNCs are collected in the Supporting Information (SI). Figure S1 collects spectra obtained in the fluorometric characterization of IrNCs, PtNCs, and AuNCs. The MNCs were previously characterized by high-resolution transmission electron microscopy, which yielded spherical shapes and average diameters of 1.9 nm for IrNCs,^23^ 1.5 for PtNCs,^24^ and 2.7 nm^25^ for AuNCs. The synthesis of the MNC-labeled immunoprobes was carried out using protocols that had also been previously optimized^23–25^ and are summarized in the SI. ICP-QMS operating conditions for MNC characterization are listed in Table 1.

#### Proteomic Analysis of EVs by Molecular Mass Spectrometry

For proteomic analysis, EVs from the four animal cohorts under study were purified from mouse blood serum initially based on a previous protocol.^28^ Briefly, serum was diluted 1:15 with cold 1× PBS, and larger particles (such as cell fragments and microvesicles) were removed by centrifugation at 40,000*g* for 2 h. The supernatant was then subjected to ultracentrifugation at 100,000*g* for 2 h, followed by a final washing of the EV pellet with 1× PBS at 100,000*g* for 2 h. All centrifugation steps were performed at 4 °C. In the present study, the isolation protocol was optimized compared to the previous version to minimize the contribution of serum proteins to the final measurements by incorporating additional washing steps for the EV pellet (details of the optimization procedures, along with a summary of the experimental results, are provided in the SI, including Table S1). The effectiveness of these studies was assessed based on the number of proteins identified during proteomic characterization for which commercial mouse serum was employed. The pellet obtained from the isolation was frozen directly at −20 °C without resuspension until further use.

The preparation of samples for proteomic analysis by LC-MS/MS began by dissolving the proteins in the EV pellet in 40 μL of a 0.2% RapiGest SF solution in 50 mM ammonium bicarbonate. For such a purpose, the solution was sonicated in 90 s cycles with 30 s pauses between cycles on ice to avoid overheating until complete dissolution of the protein pellet. Next, the total protein content was measured with the Qubit kit to determine the appropriate reagent amounts for the subsequent protocol stages. Once the protein content was determined, the following steps were carried out (amounts of reagents expressed for 50 μg of protein in 40 μL of RapiGest SF solution): (1) Addition of 4.4 μL of 50 mM dithiothreitol in 50 mM ammonium bicarbonate followed by incubation at 60 °C for 30 min; (2) Addition of 5.3 μL of 100 mM iodoacetamide in 50 mM ammonium bicarbonate followed by incubation at room temperature (RT) for 30 min, protected from light; (3) Adjustment of the pH of the solution to be within the range of 8–8.5; (4) Addition of 11.6 μL of a trypsin solution in 50 mM ammonium bicarbonate ensuring a final trypsin concentration 40 times lower than the total protein content and incubation at 37 °C for 2 h; and (5) Addition of 11.6 μL of the same trypsin solution followed by incubation at 37 °C for 15 h.

After protein digestion, the solution was prepared for LC-MS/MS analysis. To adjust the pH to 2, 2.13 μL of a 10% trifluoroacetic solution was added, and the mixture was incubated at 37 °C for 1 h. The solution was then centrifuged at 20,000*g* for 10 min, and the supernatant was transferred to a new vial, where 2.25 μL of acetonitrile was added. The solution was centrifuged again at 20,000*g* for 3 min, followed by centrifugation at 20,000*g* for 10 min. Finally, the resulting solution was adjusted with water to achieve a final protein concentration of 500 μg mL^–1^ and subjected to further analysis.

#### Competitive Immunoassay for Protein Detection in EVs

The quantitative analysis of proteins of interest in purified EVs was performed using multiplexed competitive immunoassays with MNC-labeled immunoprobes. The protein standards (ACTN, LG3BP, and MSN) used for coating and the antimouse-protein antibodies (Anti-m-ACTN, Anti-m-LG3BP, and Anti-m-MSN) were maintained at the same working concentrations across all the experiments: 26 and 20 nM, respectively. These concentrations were chosen based on previous experience with this type of immunoassay format.^29^ The immunoassays were carried out as follows: (1) Coating step: each well was coated with 100 μL of a 26 nM protein solution and incubated at 37 °C for 2 h in a microplate thermomixer; (2) Blocking step: a 1% BSA solution in 1× PBS (200 μL per well) was added and incubated at 37 °C for 2 h; (3) Washing steps: three washes with 1× PBS containing 0.1% Tween-20 were performed to remove excess blocking solution; and (4) The competitive immunoassay step was carried out. First, protein standard solutions were prepared with concentrations from 1 nM to 10^–8^ nM through serial dilutions from a 100 nM stock solution. Then, both standard solutions and EV samples (prediluted appropriately) were mixed with a 1:2 molar ratio with the MNC-labeled immunoprobe solutions (Anti-m-ACTN:AuNCs, Anti-m-LG3BP:PtNCs, and Anti-m-MSN:IrNCs) to achieve a final Ab concentration of 20 nM. The mixture was stirred at RT for 15 min in a thermomixer. Then, the prepared solutions were loaded into the coated microplate wells, and the immunoassay was performed at 37 °C for 2 h in a plate thermomixer. Five final washes with 1× PBS containing 0.1% Tween-20 were performed, and the well contents were collected with 50 μL of concentrated nitric acid and incubated for 30 min, followed by digestion in an ultrasound bath for another 30 min.

It is important to note that the three target proteins (ACTN, LG3BP, and MSN) are located within the EV lumen. Therefore, a prior lysis step was required to expose EV content to the MNC-labeled immunoprobes. This step was optimized by evaluating different concentrations of Triton X-100 (TX100) before a competitive immunoassay. A final concentration of 0.1% TX100 was added to both the protein standards and EVs samples, followed by incubation at 37 °C for 15 min prior to the competitive step. To assess potential nonspecific interactions, a negative control was included under the same competitive immunoassay conditions. In this control, a goat antihuman immunoglobulin E antibody (specific to a protein from a different species and unrelated to the targets under study) was used as the detection antibody for each of the proteins and associated MNC labels tested.

#### Determination of Target Proteins in EVs by ICP-MS

The target proteins were quantified by measuring with ICP-MS the levels of the metals in the MNC-labeled immunoprobes (IrNCs, PtNCs, AuNCs) that interacted with the coated protein in the wells after the competitive step of the immunoassay. Thus, the more element detected in the well, the less protein there is in the analyzed samples. This quantification is performed in a similar way to other calibrations, by substituting the analytical signal (amount of element in the well) in the equation of the response curve and obtaining a concentration of protein in the sample (as long as the sample concentration is in the linear region of the curve). Thus, external calibrations were performed using Ir, Pt, and Au standards (with metal concentrations up to 10 μg L^–1^ and containing 5 μg L^–1^ of Re as internal standard, IS). Specific ICP-ToFMS measurement conditions are listed in Table 1. To reduce the acidity of the solution and ensure metal levels within the external calibration range, the digested well contents were diluted 1:100 with deionized ultrapure water before ICP-MS analysis.

#### Data Normalization

For normalization of ACTN, LG3BP, and MSN concentration values, three data normalization strategies were studied in the present work. The first method was the use of the serum volume of the studied samples, determined after blood centrifugation by weighing. Another method of normalization was the total protein content of the EVs purified from serum determined with a commercial BCA kit.

We also proposed a normalization of the concentration of the target protein levels in the samples with a concentration of CD81, an EV marker protein. CD81 levels were determined with an independent competitive immunoassay, following identical experimental conditions to those explained in “Competitive Immunoassay for Protein Detection in EVs”, using anti-m-CD81:AuNCs as detection immunoprobe and not performing EV lysis, as CD81 is a protein located exclusively on EV membranes.

#### Statistical Analysis in Molecular and Elemental MS Measurements

For protein identification in proteomic analysis by LC-MS/MS, MSFragger, FragPipe, and ProteomeDiscoverer software were used. Statistical analysis of the data obtained from the different study groups by ICP-MS was performed using Origin Pro (OriginLab Corporation). Each study group included five biological replicates, except for the heterozygous Zn-supplemented individuals (+/– w/o Zn), for which only three biological replicates were available. Considering the low number of individuals per sample group, differences in protein levels between groups were evaluated using Kruskal–Wallis ANOVA. Multiple comparisons were adjusted using the Bonferroni correction.

## Results and Discussion

### Proteomic Analysis of EVs Purified from APP/PS1 Blood Serum

EVs of serum pools of male mice for each of the four cohorts under study of the APP/PS1 model were isolated following an optimized protocol with three-washing steps (the detailed optimization process of EV isolation is collected in the SI). Figure 1 depicts a summary of the obtained proteomes, represented as a Venn diagram (a detailed list of identified proteins can be found in the SI as an independent .xlsx file named *Identified proteins_APP-PS1_16-month-male_4 groups_1 replicate*). A similar number of proteins were identified in the four groups (385, 597, 475, and 594 proteins identified in the +/+ w/o Zn, +/+ w Zn, +/– w/o Zn, and +/– w Zn groups, respectively), with a slightly lower number of proteins identified in the +/+ w/o Zn group. As expected, the identity of serum proteins was similar to that previously observed in commercial mouse serum.

**Figure 1:**
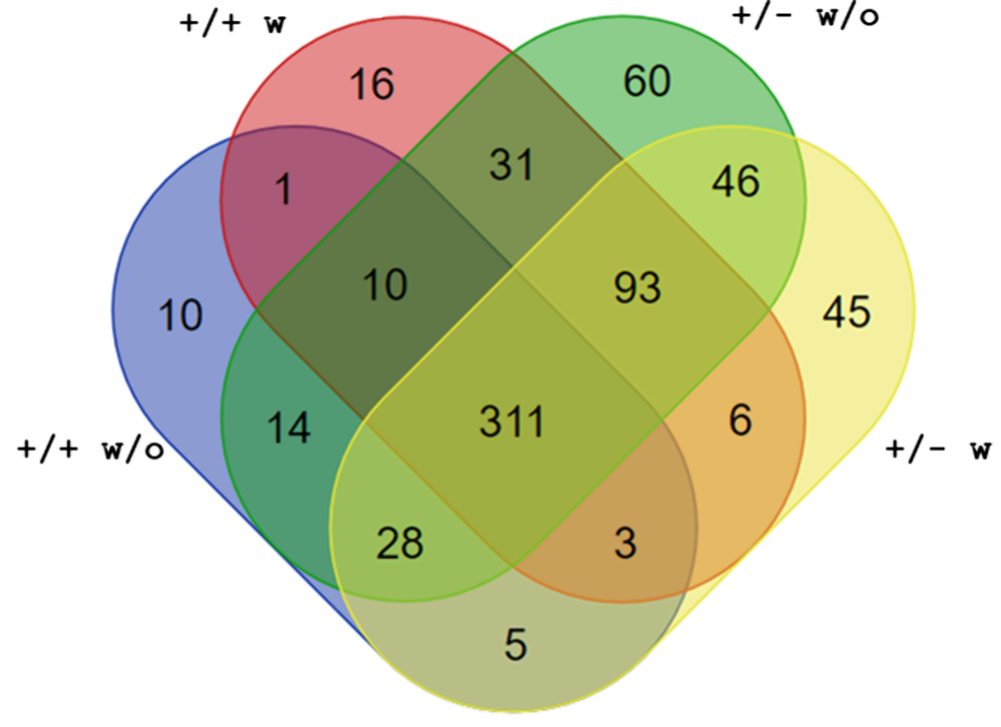
Venn diagram showing LC-MS/MS proteomic data of EVs from serum of male APP/PS1 mice (+/+ and +/–, with or without Zn supplementation). One replicate of serum pools per each group, all individuals being 16-month-old male mice, were analyzed. A detailed list of identified proteins in each sample is provided in the SI.

To identify target proteins relevant to neurodegeneration, the proteins present in the four proteomes were analyzed in detail. For this purpose, the “Top 100 Extracellular Vesicle Markers” from Vesiclepedia (http://microvesicles.org/, a bibliographic tool that compiles the most frequently reported proteins in EVs based on scientific publications) were evaluated. 41 EV markers from this list were identified among 311 common proteins in our animal model. Subsequently, a protein screening (according to the unique and total peptide values obtained in the analysis) was carried out within each group and compared with the proteome of commercial serum EVs to identify proteins showing the greatest variation between the different study groups. It is important to note that these analyses are not quantitative, as protein abundances (based, in this case, on the total and unique peptides registered) depend on the sample and experimental pretreatment. Only proteins showing substantial differences in abundance between groups were selected, with abundances normalized to the total number of proteins identified in each study group.

Finally, a bibliographic review was conducted to assess the relevance of the proteins that showed the greatest variation in the context of neurodegeneration. Based on this analysis, the following proteins were selected as targets for the development of the multiplex immunoassay for protein determination by ICP-MS: ACTN, LG3BP, and MSN. These three proteins participate in cytoskeleton dynamics and cell adhesion, closely linked to the onset of neurodegeneration. ACTN protein is intimately related to neurodegeneration onset because it tends to bind to other proteins and form aggregates, thus compromising the actin skeleton stability and cell integrity.^30^ In the case of LG3BP, the relationship of this protein with the metabolism of beta-amyloid peptide has been previously reported; elevated levels of LG3BP have been found in different samples of interest in neurodegeneration situation, so we could expect a deregulation of this protein in EVs of our working samples.^31–33^ MSN participates in the binding of the cell actin cytoskeleton to the membrane, regulating the shape of the local areas of the cell cortex. In the context of Alzheimer’s disease and neurodegeneration, it has been observed that this protein is related to the stabilization of the actin cytoskeleton mediated by Tau protein, and the consequent stabilization of neurodegenerative processes.^34^

### Optimization of the Multiplexed Immunoassay for the Detection of the Target Proteins in EVs by ICP-MS

The next step was the development of a competitive immunoassay for the simultaneous quantitative analysis of ACTN, LG3BP, and MSN in EVs from the mouse model by ICP-ToFMS. Based on the identification carried out in the proteomic study for the EVs of the mouse model and considering the average signal amplification provided by the MNCs labels (1760 for IrNCs, 1264 for PtNCs, and 466 for AuNCs^23,24,35^ ), the following protein-MNC associations were established: IrNCs for MSN, PtNCs for LG3BP, and AuNCs for ACTN detection. As can be seen in Figure S2 in the SI, the simultaneous detection of the three proteins by ICP-MS, under the operating conditions detailed in the Experimental Section, resulted in response curves with the expected fits (sigmoidal curves with negative slopes). The response curves for the target proteins yielded limits of detection (LoDs), determined considering standard deviations of each concentration tested,^36^ in the low femtomolar range.

To determine the levels of the target proteins in the EV samples, a prior lysis step is required to expose the EV lumen, as ACTN and LG3BP are exclusively present within the lumen, while MSN is present in both the EV membrane and lumen. For this purpose, TX100, a surfactant widely used for cells lysis and previously reported to be effective for EVs,^37^ was chosen. To evaluate the effectiveness of TX100 lysis, the levels of the target proteins in an EV sample from the animal model were determined by ICP-MS, comparing four final TX100 concentrations: 0.001%, 0.01%, 0.1%, and 1% (v/v). The results (Table 2) showed that increasing TX100 concentration up to 0.1% led to higher detected levels of ACTN, LG3BP, and MSN in EV samples together with a better reproducibility between replicates, while no significant changes were observed between 0.1% and 1% TX100. All tests were performed on the same sample to avoid biological variability, and the immunoassay response curves were carried out in the absence of TX100 (Figure S2).

**Table 2:** Concentration of Target Proteins (ACTN, LG3BP, and MSN) Found in EVs Purified from Blood Serum of the APP/PS1 Animal Model (16-Month-Old Heterozygous Male Nonsupplemented with Zn) Determined by ICP-MS Using the MNC-Labeled Immunoprobe Methodology and Comparing Four TX100 Concentrations for EV Lysis^a^

	[Protein] (pM)		
TX100 (%, v/v)	ACTN	LG3BP	MSN
0.001	<LoD	<LoD	4 ± 1
0.01	2 ± 1	3 ± 1	8.6 ± 0.9
0.1	7.4 ± 0.5	50 ± 4	15 ± 1
1	7.3 ± 0.9	50 ± 6	15 ± 3

a LoD stands for limit of detection, and standard deviations correspond to the measurements of three instrumental replicates per sample.

Once the optimal TX100 concentration was selected for EV lysis, a detailed study was performed to assess the effect of TX100 on immunogenic reaction. To this end, two independent immunoassays were performed for each protein studied: one in which standards were incubated with 0.1% TX100 for 15 min (“immunoassay with TX100”) and another identical immunoassay in which this step was omitted (“immunoassay without TX100”). As an example, Figure 2 shows the response curves for LG3BP detection using the Anti-m-LG3BP:PtNCs immunoprobe (response curves for ACTN and MSN, obtained using, respectively, Anti-m-ACTNP:AuNCs and Anti-m-MSN:IrNCs immunoprobes, are shown in Figure S3 of the SI). The results indicate that TX100 had a slight effect on the response curves for LG3BP and MSN (Figure 2 and Figure S3A, respectively) and a negligible effect for ACTN (Figure S3B). In all three cases, the levels of Ir, Pt, and Au detected by ICP-MS were slightly lower in the immunoassay with TX100 compared to the one without it. However, LoDs obtained were significantly lower for the three proteins when TX100 was used (37, 13, and 79 fM for MSN, LG3BP, and ACTN, respectively) compared to the immunoassay without TX100 (48, 20, and 130 fM for MSN, LG3BP, and ACTN, respectively). The methods demonstrated acceptable reproducibility with relative standard deviations (RSD) below 5% across triplicate measurements (*n* = 3) for each protein concentration tested.

**Figure 2:**
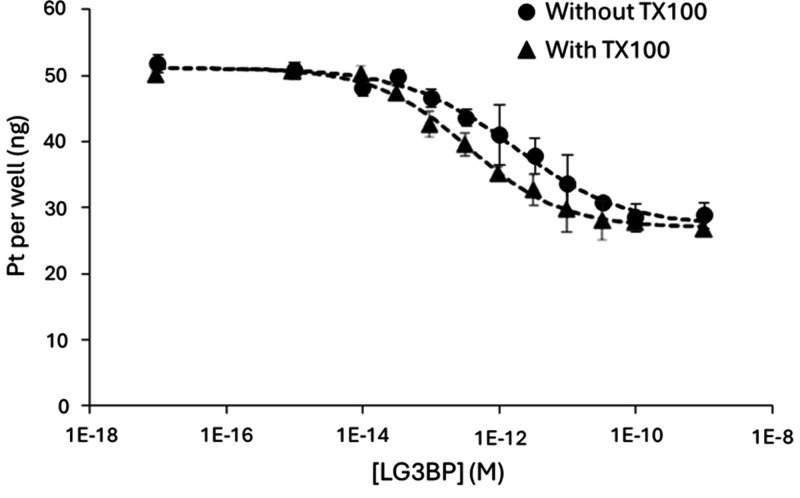
Comparative response curves obtained by ICP-MS for the competitive immunoassay for LG3BP detection using the Anti-m-LG3BP:PtNCs immunoprobe. The effect of Triton X100 (TX100) was evaluated; the dashed curve with triangles represents the response curve with TX100, whereas the dashed curve with dots represents the response curve without TX100. Error bars indicate the standard deviation of the mean signal from three independent measurements.

To confirm these results, a model sample (LG3BP, MSN, and ACTN protein standards with a known concentration of 1 pM) was analyzed using immunoassays performed with and without TX100 (Figure 2 and Figure S3). As shown in the results presented in Table 3 – Section A, the concentrations of LG3BP, MSN, and ACTN in the sample reached the expected values in all cases, with a relative error below 10%. Thus, it can be concluded that TX100 matching calibration for both standards and samples allows for accurate protein determination using the proposed methodology. However, when the study was conducted using non-TX100 matched calibrations (i.e., a sample treated with TX100 analyzed using the immunoassay without TX100 or a sample without TX100 analyzed using the immunoassay with TX100, as shown in Table 3 – Section B), the measured protein concentrations deviated significantly from the expected values. This discrepancy was particularly notable for the sample treated with TX100 when the calibration was performed without TX100, yielding ACTN, LG3BP, and MSN concentrations of 1.4 ± 0.1, 4.1 ± 0.5, and 5.7 ± 0.5 pM, respectively. These results confirmed that 0.1% TX100 is necessary in both standards and EV samples to ensure an optimal assay performance. Finally, once the immunoassay conditions were optimized, the negative control experiments performed for each target protein showed negligible nonspecific interactions between the antibodies and either the EVs or the well surfaces. The resulting Ir, Pt, and Au signals were comparable to those obtained at the highest protein concentration calibration points, where no free (unbound) antibody remained in the well, thus supporting the specificity of the method. Nonspecific interactions coming from unconjugated reagents are not considered, assuming that three washing steps of the immunoprobe synthesized would remove any unconjugated reagent’s excess.^38^

**Table 3:** Protein Concentrations Determined by ICP-MS in a Model Sample (1 pM ACTN, LG3BP, and MSN Prepared from Protein Standards) Using the Competitive Immunoassay with MNC-Labeled Immunoprobes with and without Triton X-100^a^

Section A			
	ACTN	LG3BP	MSN
Immunoassay with TX100 (Standards and Sample)			
[Protein] (pM)	1.08 ± 0.06	1.1 ± 0.1	1.02 ± 0.08
Immunoassay without TX100 (Standards and Sample)			
[Protein] (pM)	1.0 ± 0.1	1.05 ± 0.09	1.04 ± 0.08

Section B			
	ACTN	LG3BP	MSN
Immunoassay without TX100 Sample with TX100			
[Protein] (pM)	1.4 ± 0.1	4.1 ± 0.5	5.7 ± 0.5
Immunoassay with TX100 Sample without TX100			
[Protein] (pM)	0.8 ± 0.1	0.34 ± 0.03	0.20 ± 0.02

a Section A: Matched calibrations; Section B: Non-matched calibrations. Standard deviations correspond to the measurements of three instrumental replicates per sample.

### Determination of the Three Target Proteins in Serum EVs Isolated from APP/PS1 Mice by ICP-MS

Once the protocol was optimized for protein detection in EVs by a multiplexed competitive immunoassay using MNC-labeled immunoprobes and ICP-ToFMS, the analysis of the three target proteins (ACTN, LG3BP, and MSN) in EVs from the mouse model was attempted across the four cohorts described in the Experimental Section, including both male and female specimens to assess gender influence. EV isolation was carried out in an interleaved manner, alternating samples from different groups and sexes, to minimize operational biases. Each study group included five samples, except for heterozygous males and females supplemented with Zn, where only three individuals were available. In all cases, three replicates were analyzed for each sample.

For normalization purposes, three strategies were evaluated: (1) serum volume as the reference method, (2) levels of the membrane EV marker CD81, determined by an independent competitive immunoassay using a AuNC-labeled immunoprobe (Anti-m-CD81:AuNCs) and ICP-MS detection, and (3) total protein levels in the EV samples, determined by the BCA assay. As a preliminary treatment of the results obtained, sex was not considered as a variable, so that males and females in each of the study cohorts were treated as a single group. Figure S4 shows box plots for the determination of ACTN, LG3BP, and MSN in all mice analyzed. As shown in the box plots, when data for male and female mice were analyzed together, a high interindividual variability was observed; overall, the high data dispersion hindered the identification of an optimal normalization approach. In addition to the inherent biological heterogeneity of living systems, sex-based variability could affect the results. Therefore, we concluded that analyzing male and female data separately may help reduce variability and allow for a better evaluation of the normalization strategies.

When the data is segregated by sex, the trends observed allow one to draw firmer conclusions. Figure 3 shows comparative box plots for the determination of ACTN, LG3BP, and MSN in all male mice analyzed (data for female mice, with the same *y*-axis scale, are provided in the SI, Figure S5). A similar trend was observed in our experiments when comparing protein concentrations normalized by the initial serum volume (Figure 3 – Panel A) with those normalized using the CD81 levels (Figure 3 – Panel B). However, this trend was less consistent when normalization was performed using total protein content (Figure 3 – Panel C), and this may be attributed to the uncontrolled coprecipitation of serum proteins when purifying EVs. A similar pattern was observed in female mice (Figure S5). Consequently, the total protein content was discarded as a suitable normalization method.

**Figure 3:**
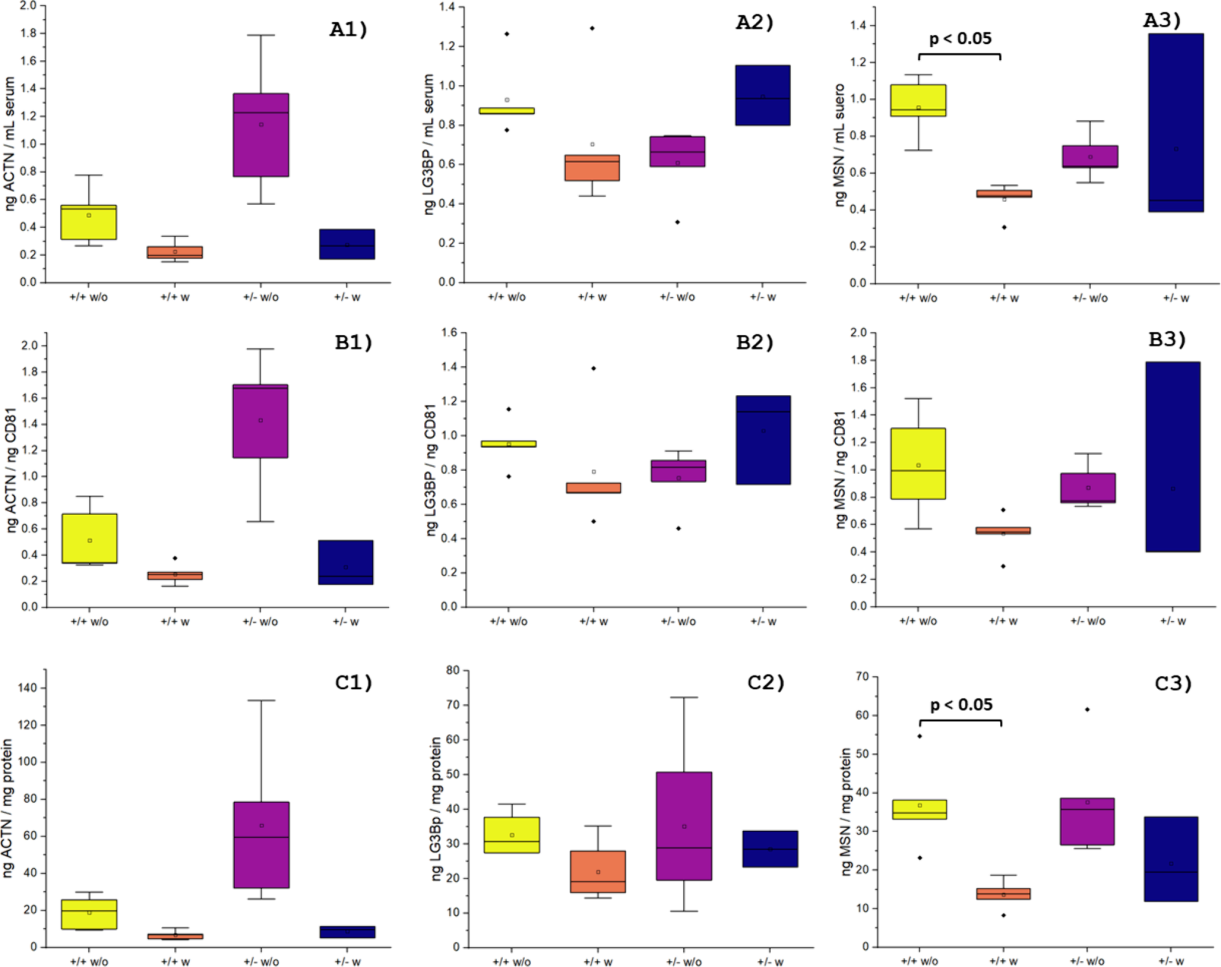
Box plots showing the normalized protein mass in EVs purified from the blood serum of male mice determined by ICP-MS for ACTN (1), LG3BP (2), and MSN (3) across four cohorts: +/+ without Zn (in yellow), +/+ with Zn (in orange), +/– without Zn (in purple), and +/– with Zn (in blue). Three normalization strategies were applied: (A) Serum volume, (B) CD81 levels, and (C) Total protein content. Data included the analysis of five individuals per group, except for heterozygous males supplemented with Zn (+/– w Zn), where only three individuals were available.

Next, the normalized average values of ACTN, LG3BP, and MSN (ng protein per mL of serum) for males and females across the different cohorts were evaluated (data available in Table S2, SI). In most cases, a clear differentiation was observed between males and females, with females generally showing lower values, especially for LG3BP and MSN. These differences could be attributed to sex-related dissimilarities in phenotypic expression,^39^ prompting the decision to additionally analyze data from both sexes separately. The trends observed for each protein are discussed in detail below.

Regarding ACTN levels in male mice (Figure 3A1), an increase in both levels and variability was observed in the +/– group with respect to the +/+ group when they were not supplemented with Zn. Conversely, in Zn-supplemented groups, ACTN levels decreased compared to their nonsupplemented counterparts, reaching similar values in both +/+ and +/– groups. This may indicate that Zn supplementation may stabilize ACTN levels regardless of disease progression. Regarding ACTN levels in female mice (Figure S5A1), no significant differences were observed between the +/+ and +/– groups in general. Zn supplementation reduced variability in the homozygous group but had no conclusive effect in the heterozygous group. This could be attributed to lower effectiveness of Zn supplementation in heterozygous females, resulting in greater data dispersion. However, no statistically significant differences (*p* > 0.05) were recorded between any of the study cohorts for both males and females.

The trends observed for LG3BP in male mice (Figure 3A2) are inconclusive. Lower levels were observed in the +/– nonsupplemented group compared to the corresponding +/+ group. However, Zn supplementation had opposite effects in both groups: while it decreased LG3BP levels in the homozygous group, it increased them in the heterozygous group. In fact, LG3BP levels in the +/+ Zn-supplemented group were comparable to those in the +/– nonsupplemented group. When comparing these results with those obtained for females (Figure S5A2), similar trends were observed, particularly the high variability in the heterozygous Zn-supplemented group (as previously noted for ACTN). These findings do not allow us to draw relevant conclusions, suggesting that LG3BP may not be a suitable biomarker for neurodegeneration in EVs. Note that, even though differences were registered in LG3BP levels between the study cohorts, no statistically significant differences (*p* > 0.05) were recorded for both male and female mice. Finally, regarding the MSN data for males (Figure 3A3), lower levels were detected in the nonsupplemented heterozygous group compared to the homozygous group, although without a statistically significant difference (*p* > 0.05). In the homozygous groups, values decreased with Zn supplementation (statistically significant with *p* = 0.01835, Kruskal–Wallis ANOVA with Dunn’s test), and this trend was also observed in the heterozygous group, though less clearly and without statistical difference (*p* > 0.05), due to the high dispersity recorded in the +/– w group. For females (Figure S5A3), the trend observed in males was repeated but in a subtler way and without statistical difference (*p* > 0.05).

## Conclusions

This study presents a multiplexed strategy for the quantification of three proteins in EVs (ACTN, LG3BP, MSN) using ICP-ToFMS and MNC-labeled immunoprobes, addressing a key challenge in EV analysis. The levels of CD81 in the isolated samples allow us to reflect EV presence, and in addition, it could act as a better alternative rather than total protein content for normalization purposes. However, it must be taken into account that CD81 concentrations can be affected by EV size and supplementation, thus restricting its applicability.

Implementation of single-EV strategies could address population-level variability and significantly enhance biomarker research. To this end, future experiments will focus on single-vesicle analysis to enable the characterization of individual EVs, thereby overcoming the averaging effects inherent to bulk measurements.

Despite the small number of animals per cohort and the fact that Zn intake was not individually controlled, preliminary results suggest that the effects of Zn supplementation and genotype may be reflected in ACTN and MSN levels, with MSN showing significant differences in specific conditions. Furthermore, although absolute protein levels were found to be different between sexes, similar trends for LG3BP and MSN were observed across study groups in both males and females, indicating a likely sex-independent regulation mechanism linked to neurodegeneration and the Zn effect for these two proteins specifically. However, these results are preliminary, as we stated before, and a proper statistical study with more individuals per group should be carried out for firmer conclusions.

Finally, this method offers a powerful alternative for EV relative protein assessment, enabling highly multiplexed protein analysis with good sensitivity. These advantages position ICP-ToFMS coupled with MNC-labeled immunoassays as a powerful tool for research in neurodegeneration and other clinically related topics.

## Supplementary Material





## Notes

Detailed list of identified proteins in each replicate of the protocols studied (ZIP)
List of reagents along with detailed protocols for the synthesis and characterization of MNCs and MNCs-labeled immunoprobes (Ab:MNCs); information on the optimization of EV purification for protein selection via LC-MS/MS as well as the resulting proteomes for all samples; response curves for the three MNC-labeled immunoprobes used in the multiplexed competitive immunoassay (targeting MSN, LG3BP, and ACTN), accompanied by comparative immunoassay responses with and without TX100; box plots showing the protein mass in EVs purified from the blood serum of all mice (both, male and female), as well as from female mice only, across the four cohorts; comparative table summarizing the average protein mass values normalized to serum volume for male and female mice (PDF)
