# Supplementary material for: ICP-MS for Multiplexed Protein Determination in Extracellular Vesicles from APP/PS1 Mice Blood Serum–Application to a Zn Supplementation Pilot Study
Source: Anal Chem. 2025 Sep 15;97(38):20928–36. doi: 10.1021/acs.analchem.5c03630 (PMC12489885; doi:10.1021/acs.analchem.5c03630)
Supplement: Supplementary file 2 [file ac5c03630_si_002.pdf]

SUPPORTING INFORMATION

ICP-MS for Multiplexed Protein Determination in Extracellular Vesicles from APP/PS1 Mice Blood Serum —Application to a Zn Supplementation Pilot-study

Jaime Martínez-García[1], Beatriz Fernández[1], Enol Artime[2], Lidia Álvarez[2], Héctor González-Iglesias[3], David Clases[4], Rosario Pereiro[1]

[1]*Department of Physical and Analytical Chemistry, University of Oviedo, Julian Clavería 8, 33006 Oviedo, Spain.*

[2]*Fundación de Investigación Oftalmológica (FIO), Avda. Dres. Fernández-Vega, 34, 33012 Oviedo, Spain*

[3]*Dairy Research Institute of Asturias, Spanish National Research Council (IPLA-CSIC), C. Francisco Pintado Fe, 26, 33011 Oviedo, Spain*

[4]*NanoMicroLab, Analytical Chemistry, Institute of chemistry, University of Graz, Universitätsplatz 1, 8010, Graz, Austria.*

**Corresponding author: fernandezbeatriz@uniovi.es*

ABSTRACT

The Supporting Information (SI) contains details related to the reagents employed, the protocols referenced in the *Experimental Section*, and information for the *Results and Discussion Section*. A complete list of the reagents used is collected below. The protocols for the synthesis and characterization of metal nanoclusters (MNCs) and MNCs-labelled immunoprobes (Ab:MNCs) are then described. Details of the characterization of the MNCs synthesized are also collected. For the selection of the proteins of interest using LC-MS/MS, information about extracellular vesicles (EVs) purification optimizations using commercial mouse serum, and the proteomes obtained for all samples, are depicted. Regarding the immunoassay, response curves for the three MNCs-labelled immunoprobes used in the multiplexed competitive immunoassay to detect Moesin (MSN), LG3BP and Actinin (ACTN) by ICP-MS are provided. These curves are accompanied by comparative responses of the immunoassay obtained with and without Triton X-100 (TX100). Moreover, box plots showing the protein mass in EVs purified from the blood serum of all mice (both, male and female) and just female mice determined by ICP-ToFMS for ACTN, LG3BP and MSN across the four cohorts are included. Finally, a comparative Table with average protein mass values normalized to serum volume for male and female individuals is also provided.

EXPERIMENTAL SECTION

Reagents

EVs were isolated from mouse serum with a differential centrifugation-based protocol, for which 4 mL open-top thin wall polypropylene ultracentrifuge tubes (Beckman Coulter, Ref. 328874) were used. For serum dilution and EVs resuspension, freshly prepared and filtered 1X phosphate buffered saline (PBS) pH 7.4 was employed. A filtration step of the EVs suspension using Millex-GV 0.22 µm low protein binding Durapore® polyvinylidene difluoride (PVDF) filtration units (Merck Millipore, Ref. SLGV013SL) was performed before the washing steps. Total protein content in isolated serum EVs was determined with the bicinchoninic acid assay using a Pierce™ BCA protein assay kit (Thermo Fisher, Ref. 23227). For procedural optimization purposes, commercial mouse serum (VWR, Ref. S2160-050) was applied as source of EVs.

For the synthesis of MNCs, the following reagents were employed: iridium (III) chloride hydrate (Alfa Aesar, Ref. 11030), chloroplatinic acid solution (8% (w/v) in water) (Sigma

Aldrich, Ref. 262587), sodium tetrachloroaurate (III) dihydrochloride (Sigma Aldrich, Ref. 298174), tri-sodium citrate dihydrate (Merck, Ref, A385148), DL-thioctic acid (Acros Organics, Ref. 138720050), sodium hydroxide (Sigma Aldrich, Ref. 06203), sodium borohydride (Sigma Aldrich, Ref. 71320) and Alphagaz™ 1 Nitrogen gas (Air Liquide, Ref. P0271L50S2A001). For the conjugation of MNCs to antibodies, the crosslinking agents 1-ethyl-3-(3-dimethylaminopropyl)carbodiimide (EDC) hydrochloride (Sigma Aldrich, Ref. 03450) and N-hydroxysuccinimide (NHS) (Sigma Aldrich, Ref. 56480) were used. For the purification of both MNCs and immunoprobes, Amicon® ultra centrifugal filters (3 kDa and 100 kDa, respectively) (Merck Millipore, Refs. UFC500324 and UFC510024) were used. For the blocking of the immunoprobes, hydroxylamine hydrochloride (Sigma Aldrich, Ref. 159417) was utilized.

Sample preparation for proteomic analysis by molecular MS was performed using the following reagents: RapiGest surfactant (Water, Ref. 186001860) solution (0.2% (v/v) in 50 mM ammonium bicarbonate, Qubit™ protein assay kit (ThermoFisher Scientific, Ref. A50668), dithiothreitol (DTT) (Sigma Aldrich, Ref. D0632), iodoacetamide (Sigma Aldrich, Ref. 407710), sequencing grade modified porcine trypsin (Promega, Ref. V5111). For tandem MS analysis, previously digested EVs samples were treated by adding trifluoroacetic acid (Sigma Aldrich, Ref. 302031) 10 % (v/v) solution and acetonitrile (Sigma Aldrich, Ref. 34851).

For the immunoassays developed in this work, clear flat-bottom immuno 96-well plates (ThermoFisher, Ref. 442404) were used. Lyophilized bovine serum albumin (BSA) (Sigma Aldrich, Ref. A4503) was employed for blocking. For the washing steps, the washing buffer was freshly prepared in PBS using Tween-20 (Sigma Aldrich, Ref. P2287) nonionic detergent. The following biological reagents were used in the immunoassay: rabbit Anti-mouse CD81 (Abcam, Ref. ab109201), recombinant mouse CD81 protein (UniProt code P35762) (Abcam, Ref. ab276921), rabbit Anti-mouse alpha actinin (Abcam, Ref. ab68194), alpha-actinin-1 recombinant protein (UniProt code Q7TPR4) (MyBioSource, Ref. MBS1460737), rabbit Anti-mouse LGALS3BP (Abcam, Ref. ab217572), LGALS3BP recombinant protein (UniProt code Q07797) (MyBioSource, Ref. MBS2031054), rabbit Anti-mouse moesin (Abcam, Ref. ab52490), recombinant human moesin protein (UniProt code P26041) (Abcam, Ref. ab64309), and goat Anti-human Immunoglobulin E (Sigma Aldrich, Ref. I6284). For EVs lysis, Triton X-100 (Sigma Aldrich, Ref. T8787) solutions in 1X PBS were applied.

For ICP-MS measurements, trace metal standards were prepared from 1000 mg L^{-1} standard solution of each metal (Ir, Pt, Au, Pd and Re, Sigma-Aldrich, Refs. 1703250100, 19078, 170321, 77091 and 39957, respectively) in deionized ultrapure water. For the digestion of NPs, concentrated HNO_3 solution (LabKem, Ref. NIAC-50P-1K0) previously sub-boiling distillated, and ultra-trace grade hydrochloric acid (Carlo Erba, Ref. 403915) were used. Deionized ultrapure water, resistivity 18.2 MΩ·cm (Purelab Flex 3&4; ELGA-Veolia, High Wycombe) was utilized throughout.

Synthesis of MNCs and MNCs-Labelled Immunoprobes

Metal-based (Ir, Pt and Au) NCs were synthesized using protocols previously optimized by our research group[i-iii]. Briefly, the three protocols are as follows. For IrNCs, 3 mL of a 33 mM citrate solution, 20 mL of a 1 mM $IrCl_3$ solution, and 10 µL of 2 M NaOH were sequentially mixed in a double-neck round-bottom flask under stirring and an inert atmosphere (N_2 gas bubbling through the solution). The mixture was heated to reflux conditions and maintained for 20 min. It was then cooled to room temperature (RT), after which 1 mL of a 100 mM $NaBH_4$ solution was added under stirring while maintaining an inert atmosphere. The reaction proceeded at 25 °C for 15 h. Subsequently, 60 µmol of lipoic acid was added directly to the mixture under stirring and an inert atmosphere, along with 50 µL of a 100 mM $NaBH_4$ solution. The resulting mixture was stirred for an additional 9 h. Finally, under ambient atmosphere, the pH was adjusted to 6 to ensure the long-term stability of the synthesized IrNCs.

For PtNCs and AuNCs, the synthesis protocol is largely similar. Lipoic acid (50 µmol for PtNCs and 30 µmol for AuNCs) was added to a final volume of water (10 mL for PtNCs and 20 mL for AuNCs), along with a small amount of 2 M NaOH solution (30 µL for PtNCs and 50 µL for AuNCs). The mixture was stirred in a 25 °C water bath until all reagents were fully dissolved. Next, the precursor solution (200 µL of 45 mM $PtCl_6^{2-}$ for PtNCs or 200 µL of 50 mM $AuCl_4^-$ for AuNCs) was added and mixed for 5 min. Finally, the reductant solution (400 µL of a 50 mM $NaBH_4$ solution for both syntheses) was added, and the reaction was left under stirring at 25 °C for 15 h.

MNCs must be purified before characterization and for the synthesis of MNCs-labelled immunoprobes. Amicon® 0.5 mL 3 kDa centrifugal filters were used, and three centrifugal steps at 5,000xg for 30 min each were performed. MNCs were resuspended either in water for characterization purposes, or in 1X PBS for antibody labeling.

Regarding MNCs-labelled immunoprobes, they were synthesized following previously published protocols[i-iii]. In this study, four different immunoprobes were synthesized using a specific antibody (Ab) for the target proteins: Anti-m-ACTN:AuNCs, Anti-m-LG3BP:PtNCs, Anti-m-MSN:IrNCs and Anti-m-CD81:AuNCs. In all cases, labeling was performed with a final Ab concentration of 20 μg mL^{-1}. The Ab:MNCs molar ratios were as follows: 1:10 for Anti-m-MSN:IrNCs, 1:20 for Anti-m-LG3BP:PtNCs, and 1:5 for Anti-m-ACTN:AuNCs and Anti-m-CD81:AuNCs. The crosslinking reagents EDC and NHS were used at an Ab:EDC:NHS molar ratio of 1:1500:1500. The reagents were mixed as follows: first, the Ab and MNCs solutions were mixed under stirring for 15 min. Then, the EDC/NHS solution was added, and the mixture was stirred at RT for 2 h. The resulting immunoprobes were purified using Amicon® 0.5 mL 100 kDa centrifugal filters with three centrifugal steps at 3,500xg for 5 min each. Finally, they were resuspended in 1X PBS until further use.

# Characterization of metal-based nanoclusters

MNCs exhibit photoluminescent properties, due to their ultra-small size. Thus, each of them exhibits a characteristic excitation and emission maximum. Therefore, a photoluminescent characterization of the MNCs synthesized in this work was performed. To do so, purified aliquots of the synthesis were independently introduced in a 1 mL quartz cuvette, and excitation and emission spectra were collected as follows: (1) For the excitation spectra, the emission wavelengths were fixed at 545, 620 and 710 nm for Ir, Pt and Au NCs, respectively, and the spectra were recorded from 350 to 550 nm; and (2) For the emission spectra, the excitation wavelengths were fixed at 385, 455 and 400 nm for Ir, Pt and Au NCs, respectively, and the spectra were recorded from 500 to 750 nm. Spectra were acquired at 1 nm intervals, with entrance and exit slits widths at 10 and 20 nm, respectively.

Finally, MNCs were characterized by ICP-MS to know the experimental levels of Ir, Pt and Au in the purified synthesis solution. Metals concentration is needed to estimate the particle concentration for the synthesis of the immunoprobes, and useful to know the synthesis yield and recovery. 50 μL aliquots of unpurified synthesis, purified synthesis and purification supernatants for each of the MNCs synthesized were digested with 200 μL concentrated HNO_3 (for IrNCs) or aqua regia (for PtNCs and AuNCs) in an ultrasound bath for 30 min. Then, digested samples were diluted 1:200 with water to reduce acidity of the solution, and these solutions were then diluted with 2% HNO_3 to fit external

calibrations (1:200 for unpurified and purified synthesis, 1:13 for Ir and Pt purification supernatants, and no dilution for Au purification supernatants), with 5 µg L^{-1} Pd final concentration (used as internal standard - IS). ICP-QMS operating conditions are depicted in **Table 1** of the manuscript.

RESULTS AND DISCUSSION

Characterization of metal-based nanoclusters

Once the MNCs were purified, the fluorometric characterization yielded excellent results, confirming success of the syntheses (**Figure S1**).

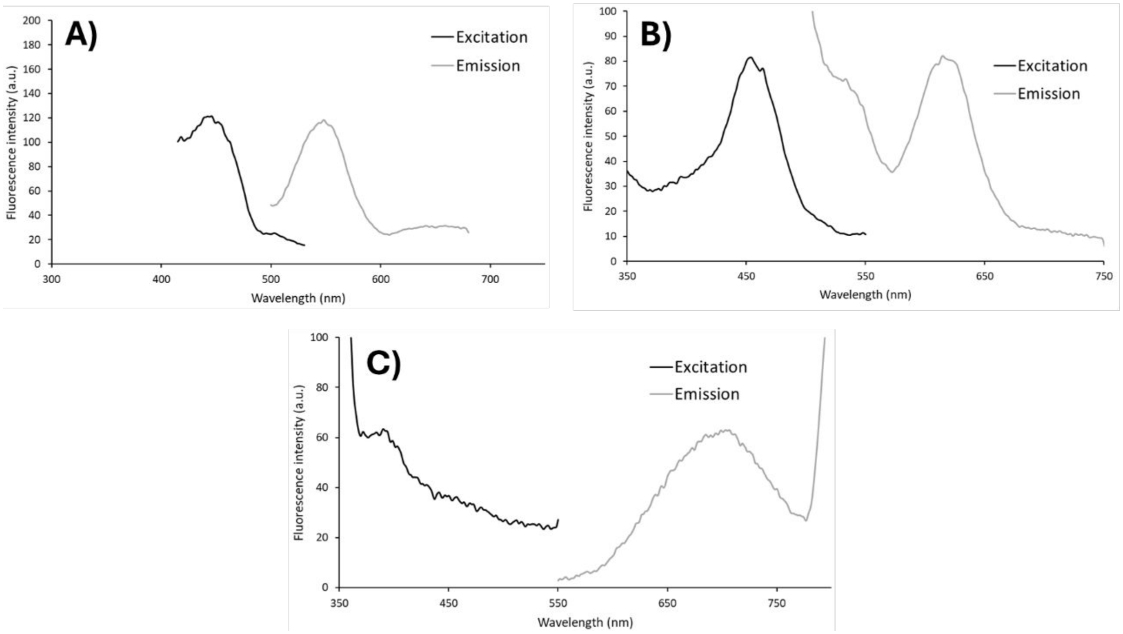

Figure S1. Experimental excitation (black) and emission (gray) spectra obtained in the fluorimetric characterization of IrNCs (A), PtNCs (B) and AuNCs (C).

ICP-QMS characterization was performed for the three synthesized MNCs in order to determine the particle concentration (data needed for the Ab:MNCs immunoprobes synthesis). The experimental diameters obtained for IrNCs, PtNCs and AuNCs were 1.9 nm, 1.5 nm and 2.7 nm, respectively[i-iii]. In addition, knowing that the crystalline structure of all three MNCs is face-centered cubic (FCC), it is known that each unit cell contains 4 atoms. Knowing the density values for the three elements studied (22.6 g cm^{-3} for Ir, 21.5 g cm^{-3} for Pt and 19.3 g cm^{-3} for Au) and mass per atom (3.19 10^{-22} g for Ir, 3.24 10^{-22} g for Pt and $3.27 \cdot 10^{-22}$ g for Au), the cell unit volume could be estimated as 5.65, 6.02 and 6.78 10^{-2} nm^3, respectively. Finally, knowing the number of unit cells per particle

(calculated by knowing the particle volume assuming spherical shape, and considering the volume per unit cell previously calculated), the average number of atoms per particle can be estimated as 250, 115 and 600 for IrNCs, PtNCs and AuNCs, respectively.

Once these mathematical calculations were made, an estimation of the molar concentration of particles in the synthesis solutions is performed, considering the experimental concentrations of the elements obtained by ICP-QMS (0.40, 0.50 and 0.35 mM for Ir, Pt and Au, respectively). With this, and the moles of element per particle (calculated with the number of element atoms per particle previously obtained), the number of particles in the solution can be estimated. Finally, knowing the synthesis volume, an estimation of the particles per unit volume is obtained as 2.1, 6.3 and 0.7 µM for IrNCs, PtNCs and AuNCs, respectively.

# Proteomic Analysis of EVs purified from APP/PS1 blood serum

For the isolation of EVs from mouse serum for immunoassay applications, a protocol based on differential centrifugation was previously optimized to achieve the highest yield of small EVs[iv], obtaining a quite homogeneous EVs size distribution, predominantly 40-50 nm diameter. However, during proteomic analysis by molecular MS of EVs from mouse blood serum, we observed that these purified EVs produced measurement artifacts. In biomarker disease research using serum-derived samples, most of target proteins are present at very low levels, and proteomic analysis of such samples is particularly challenging because abundant serum proteins (e.g., albumins, lipoproteins and immunoglobulins) can partially or completely mask the detection of the proteins of interest, thus leading to underestimated protein abundances or even failing to detect proteins that are actually present[v]. Therefore, the initial procedure was optimized by analyzing the proteome of EVs purified from commercial mouse serum, leading to the establishment of the following criterion: the contribution of abundant serum proteins in proteomic analysis is considered negligible if these proteins are either not identified or detected at relatively low abundances, while EVs marker proteins (such as tetraspanins CD9 and CD81) are successfully identified. Three protocols were evaluated, designated as one-, three- and four-washing steps.

As can be seen in **Table S1**, the initial protocol (one-washing step) detected 475, 463 and 583 proteins in each independent replicate, with 378 proteins common across all three replicates (detailed information of the identified proteins for each replicate is provided as an independent .xlsx file named *Identified proteins_commercial serum_1 wash_3*

replicates). However, none of the proposed EVs markers were identified, and most of the abundant serum proteins identified were among the top 40 proteins. These results highlight the masking effect of abundant serum proteins on target EVs proteins that are in the low-level range. To overcome this issue, a protocol involving more washes of the EVs pellet obtained from blood serum was applied. Previous studies have shown that multiple washes can reduce the contribution of abundant serum proteins in subsequent proteomic analyses without diminishing the presence of EVs in the pellet[vi].

Thus, to identify the optimal EVs isolation protocol for proteomics, we studied two alternatives: performing three or four washing steps of the EVs pellet with 1X cold PBS at 100,000xg for 70 min. As shown in **Table S1**, a significant increase in the number of identified proteins was observed compared to the one-washing step protocol: 689-698 proteins for the three-washing steps protocol and 506-626 proteins for the four-washing steps protocol compared to 463-583 for the one-washing step (detailed information for each replicate can be found as independent .xlsx files named *Identified proteins_commercial serum_3 washes_3 replicates* and *Identified proteins_commercial serum_4 washes_2 replicates*). Thus, the experimental results indicate that the masking effect caused by abundant serum proteins is less pronounced with additional washing steps in the purification protocol. Although similar number of abundant serum proteins were identified (between 28 and 32) with the three conditions tested, the abundances were much lower than in the proteomes of EVs purified with the one-washing step protocol (see .xlsx files provided for detailed information). Regarding the identification of proteins of interest, CD9 was detected in two of the three replicates purified with the three-washing steps protocol, and CD9 and CD81 were both detected in the two replicates purified with the four-washing steps protocol. However, it should be noted that the number of proteins detected between replicates with the three-washing steps protocol (three replicates, 698, 691 and 689 proteins, with 544 common proteins) was more consistent than in the four-washing steps (two replicates, 626 and 506 proteins, with 488 common proteins). Therefore, the three-washing steps protocol was selected for proteomic analysis, as it offers a better balance between proteome homogeneity across replicates and the contribution of EVs markers and abundant serum proteins.

Table S1. Summary of the results obtained in proteomic analyses for the optimization of the isolation protocol of EVs from commercial mouse blood serum. The Table shows the number of proteins detected by LC-MS/MS, the common proteins identified across the replicates per protocol, and the number of serum proteins detected in each sample. Detailed list of identified proteins in each replicate of the protocols studied are provided as independent .xlsx files.

	**Detected proteins**	**Common proteins**	**Serum proteins detected**
**One-washing step (n = 3)**	475	378	30
	463		31
	583		32
**Three-washing steps (n = 3)**	698	544	31
	691		31
	689		30
**Four-washing steps (n = 2)**	626	488	29
	506		28

A detailed list of the proteomes obtained for the samples (commercial mouse blood serum detailed in this section and male mice from the four study cohorts of the APP/PS1 model, detailed in the manuscript) analyzed by LC-MS/MS is provided as separate .xlsx files alongside this document. Data related to the optimization of the EVs isolation protocol (one-, three- and four-washing steps protocols using the commercial blood serum – as shown in **Table S1**) are compiled as *Identified proteins_commercial serum_X(1, 3 or 4) washes_X (2 or 3) replicates*. Data corresponding to the analysis of EVs isolated from blood serum of male mice from the four study cohorts of the APP/PS1 model (**Figure 1** of the manuscript) are denoted as *Identified proteins_APP-PS1_16-month-male_4 groups_1 replicate*.

# Optimization of the Multiplexed Immunoassay for the Detection of the Target Proteins in EVs by ICP-ToFMS

In the present work, a methodology based on a competitive immunoassay with ICP-ToFMS detection has been developed for the simultaneous detection of MSN, LG3BP and ACTN proteins using Ir-, Pt- and Au-based MNCs, respectively. **Figure S2** collects

the experimental response curves (amount of element in well *vs.* protein concentration) obtained in the immunoassay for each of the proteins studied.

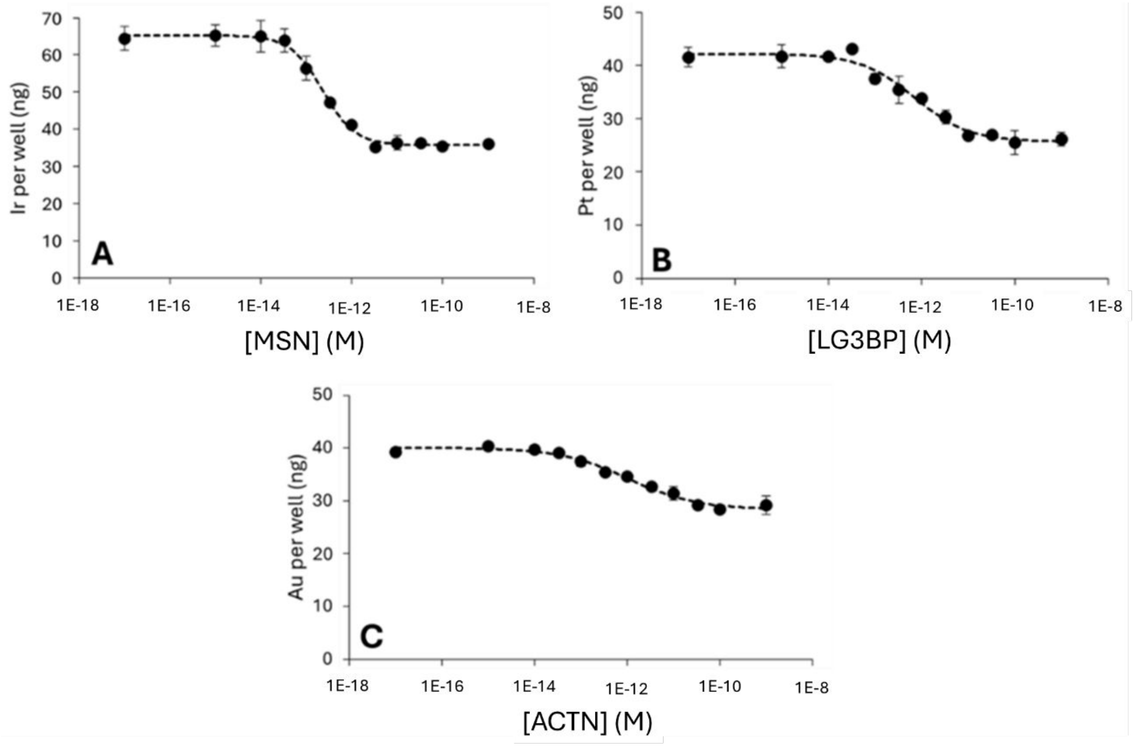

Figure S2. Response curves performed using the competitive immunoassay with MNCs as labels and ICP-ToFMS detection. A) Response curve for MSN immunoassay using Anti-m-MSN:IrNCs immunoprobe; B) Response curve for LG3BP immunoassay using Anti-m-LG3BP:PtNCs immunoprobe; and C) Response curve for ACTN immunoassay using Anti-m-ACTN:AuNCs immunoprobe. Error bars represent the standard deviation of the mean values of the signal for three independent measurements.

In addition to the previous experiments, for an adequate detection of the proteins in EVs samples, lysis with Triton X-100 (TX100 was required to expose the entire content of the EVs to the immunoprobes. In the present work, the effect of the use of TX100 on the ICP-ToFMS detection for the developed immunoassay was studied, to ensure that its use does not introduce artifacts in the detection. **Figure S3** shows the responses obtained in immunoassays with and without TX100 for MSN-IrNCs and ACTN-AuNCs, whereas the results for LG3BP-PtNCs were pictured in **Figure 2** of the manuscript.

A B

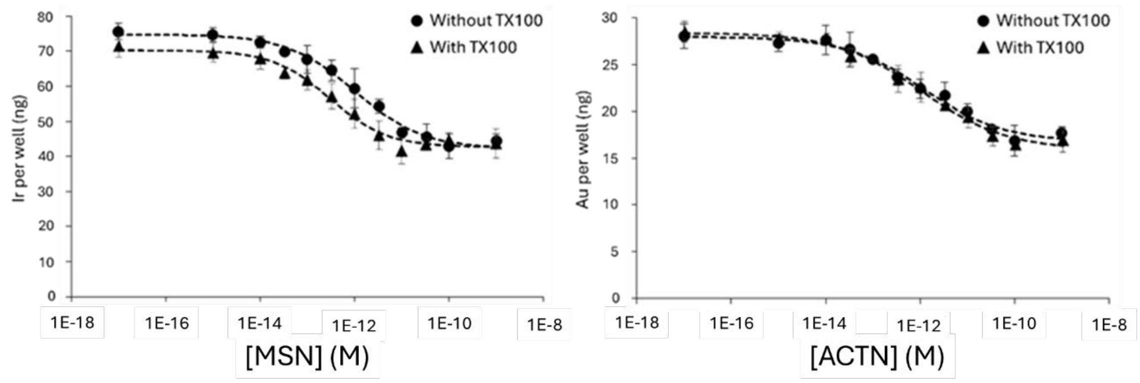

Figure S3. Comparative responses obtained by ICP-MS in the competitive immunoassay to evaluate the effect of TX100 as lysis agent in the determination of proteins in EVs. A) Response curves for MSN immunoassay using the Anti-m-MSN:IrNCs immunoprobe; and B) Response curve for the ACTN immunoassay using Anti-m-ACTN:AuNCs immunoprobe. Dashed curves with dots represent responses obtained without using TX100, while dashed curves with triangles represent responses with TX100. Error bars indicate the standard deviation of the mean signal from three independent measurements.

Determination of Three Target Proteins in Serum EVs Isolated from APP/PS1 Mouse Model by ICP-ToFMS: A Normalization Strategy.

Once the immunoassay for the simultaneous detection of ACTN, LG3BP and MSN has been developed, the values of these proteins were determined in isolated EVs of serum samples from APP/PS1 model individuals. **Figure S4** shows the box plots comparing the levels obtained for 16-month-old male and female mice, with the three normalization strategies evaluated in the present work (serum volume, CD81 concentration, and total protein). The levels referred to 16-month-old females' cohorts are reflected in **Figure S5**, and those referred to males' cohorts are reflected in **Figure 3** of the manuscript.

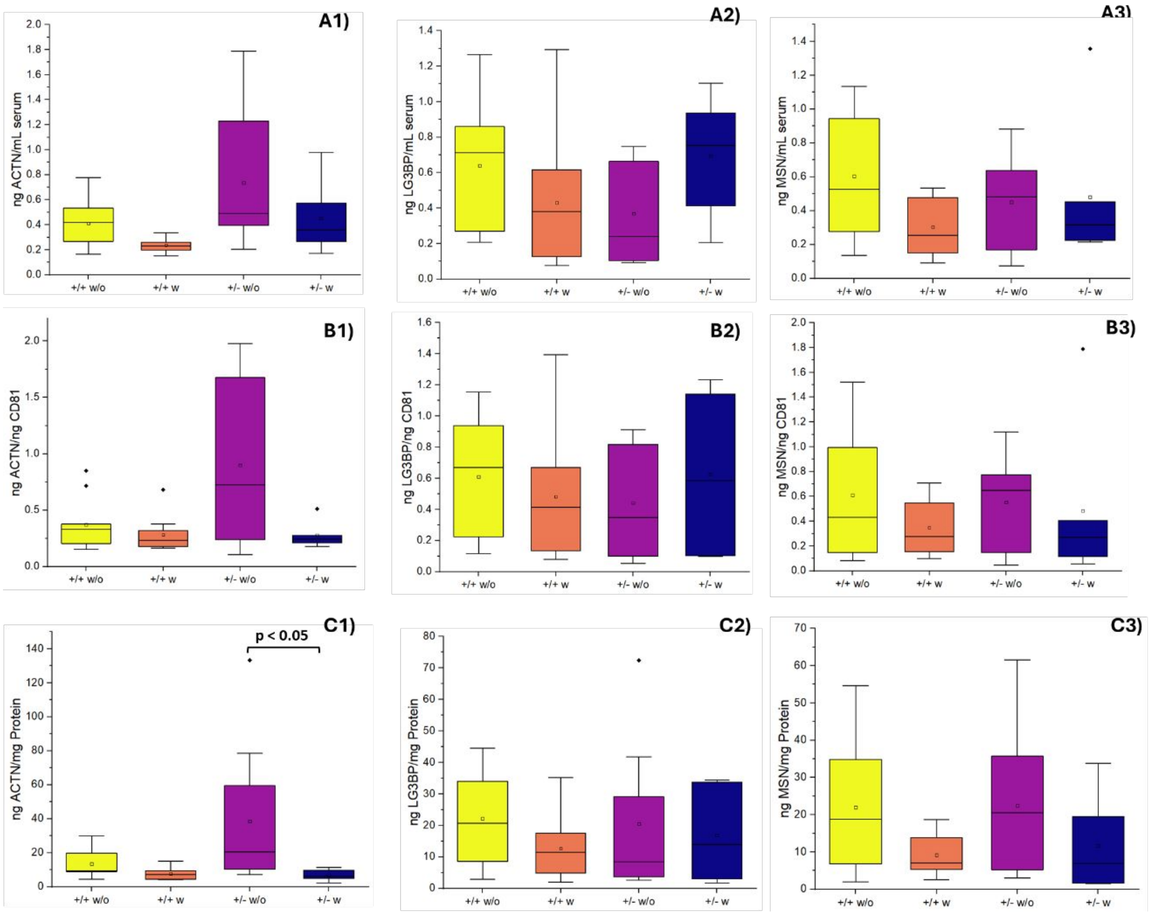

Figure S4. Box plots showing the normalized protein mass in EVs purified from the blood serum including male and female APP/PS1 mice determined by ICP-MS for ACTN, LG3BP and MSN across four cohorts: +/+ without Zn (in yellow), +/+ with Zn (in orange), +/- without Zn (in purple) and +/- with Zn (in blue). Three normalization strategies were applied: A) Serum volume, B) CD81 levels, C) Total protein content. Data included the analysis of ten individuals per group, except for heterozygous individuals supplemented with Zn (+/- w), where only six individuals were available. In all cases, three instrumental replicates were measured. For the sake of comparison, the scale of the y-axis in all graphs has been set equal to that of the graphs representing data from males' samples (**Figure 3** of the manuscript).

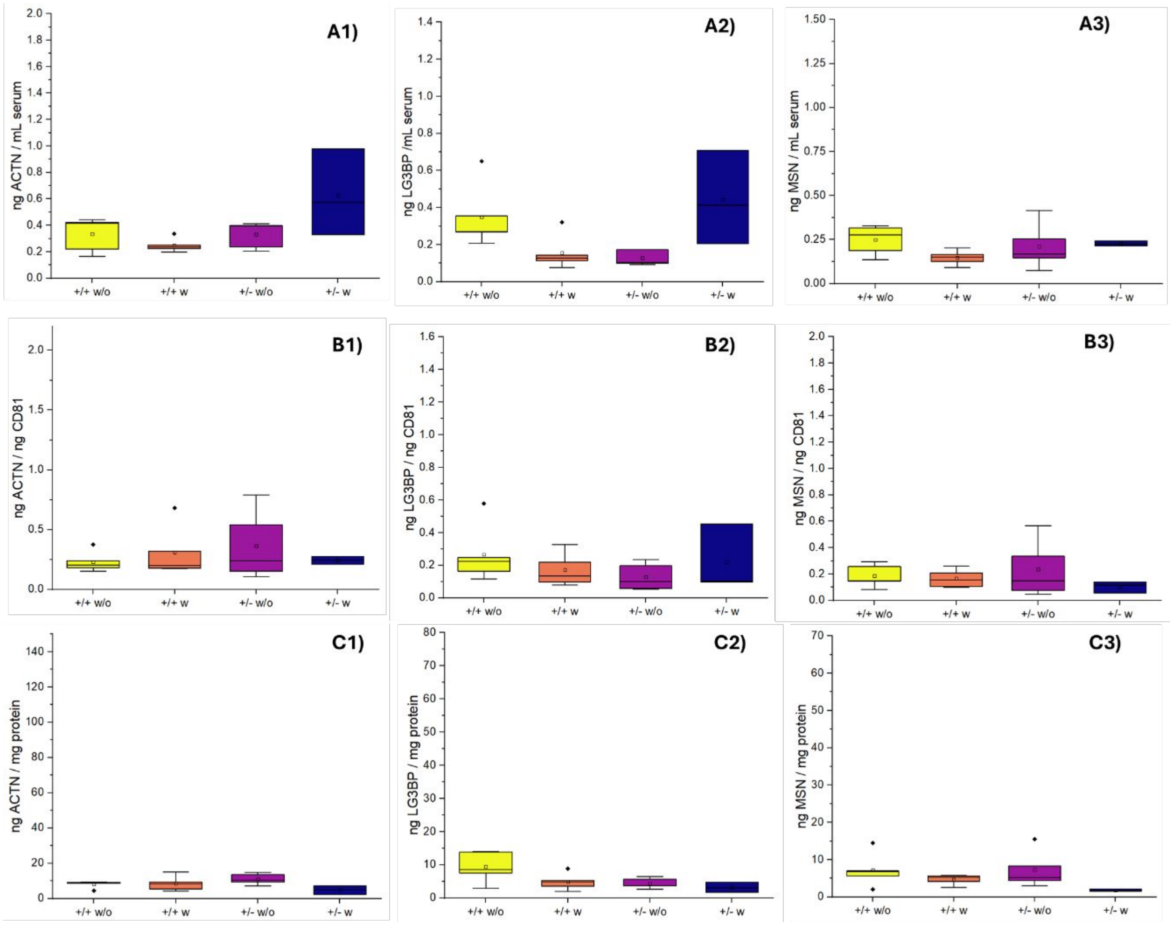

Figure S5. Box plots showing the normalized protein mass in EVs purified from the blood serum of female APP/PS1 mice determined by ICP-MS for ACTN, LG3BP and MSN across four cohorts: +/+ without Zn (in yellow), +/+ with Zn (in orange), +/- without Zn (in purple) and +/- with Zn (in blue). Three normalization strategies were applied: A) Serum volume, B) CD81 levels, and C) Total protein content. Data included the analysis of five individuals per group, except for heterozygous females supplemented with Zn (+/- w Zn), where only three individuals were available. In all cases, three instrumental replicates were measured. For the sake of comparison, the scale of the y-axis in all graphs has been set equal to that of the graphs representing data from males' samples (**Figure 3** of the manuscript).

To highlight the differences between males and females, from the box plots previously presented for males (**Figure 3** of the manuscript) and females (**Figure S5** of the present SI), the average values for each of the proteins and cohorts studied were extracted and are collected in **Table S2**.

Table S2. Average levels (and their standard deviations) of ACTN, LG3BP and MSN proteins (expressed as ng of protein per mL serum) in EVs analyzed by ICP-MS across the four cohorts, normalized by serum volume and differentiated by sex (n = 5 per group, except for +/- w/o Zn, where n = 3). Average values normalized using CD81 levels showed similar trends and are therefore not included below. Note that the deviation is quite high in some cases due to the inherent biological variability of aged animal models.

	**Average Normalized Mass of Protein (ng of protein per mL of serum)**		
**Protein**	**Group**	**Males**	**Females**
**ACTN**	**+/+ w/o Zn**	0.5 ± 0.2	0.3 ± 0.1
	**+/+ w Zn**	0.23 ± 0.08	0.25 ± 0.05
	**+/- w/o Zn**	1.1 ± 0.5	0.3 ± 0.1
	**+/- w Zn**	0.3 ± 0.1	0.6 ± 0.3
**LG3BP**	**+/+ w/o Zn**	0.9 ± 0.2	0.3 ± 0.2
	**+/+ w Zn**	0.7 ± 0.3	0.2 ± 0.1
	**+/- w/o Zn**	0.6 ± 0.2	0.13 ± 0.04
	**+/- w Zn**	0.9 ± 0.2	0.4 ± 0.3
**MSN**	**+/+ w/o Zn**	0.9 ± 0.2	0.25 ± 0.08
	**+/+ w Zn**	0.46 ± 0.09	0.15 ±0.04
	**+/- w/o Zn**	0.7 ± 0.1	0.2 ± 0.1
	**+/- w Zn**	0.7 ± 0.5	0.23 ±0.02

REFERENCES

(i) Menero-Valdes, P.; Lores-Padin, A.; Fernandez, B.; Gonzalez-Iglesias, H.; Pereiro, R. Iridium Nanoclusters as High Sensitive-tunable Elemental Labels for Immunoassays: Determination of IgE and APOE in Aqueous Humor by Inductively Coupled Plasma-Mass Spectrometry. *Talanta* **2022**, 244, No. 123424.

(ii) Lores-Padin, A.; Cruz-Alonso, M.; Gonzalez-Iglesias, H.; Fernandez, B.; Pereiro, R. Bimodal determination of Immunoglobulin E by Fluorimetry and ICP-MS by Using Platinum Nanoclusters as a Label in an Immunoassay. *Microchim. Acta* **2019**, 186, No. 705.

(iii) Cruz-Alonso, M.; Fernández, B.; Alvarez, L.; Gonzalez-Iglesias, H.; Traub, H.; Jakubowski, N.; Pereiro, R. Bioimaging of Metallothioneins in Ocular Tissue Sections by LA-ICP-MS using Bioconjugated Gold Nanoclusters as Specific Tags. *Microchim. Acta* **2018**, 185, No. 64.

(iv) Martínez-García, J.; Fernández, B.; Pereiro, R. Quantification of CD81 in Mouse Serum-derived Extracellular Vesicles via Inductively Coupled Plasma – Mass Spectrometry Using Gold-Based Labels. *Talanta* **2026**, 297, No. 128609.

(v) Fochtman, D.; Marczak, L.; Pietrowska, M.; Wojakowska, A. Challenges of MS-based Small Extracellular Vesicles Proteomics. *J. Extracell. Ves.* **2024**, 13, No. e70020.

(vi) An, M.; Wu, J.; Zhu, J.; Lubman, D. M. A Comparison of an Optimized Ultracentrifugation Method Versus Size-exclusion Chromatography for Isolation of Exosomes from Human Serum. *J. Proteome Res.* **2018**, 17, 3599-3605.
